# Adapting to climate change: responses of fine root traits and C exudation in five tree species with different light-use strategy

**DOI:** 10.3389/fpls.2024.1389569

**Published:** 2024-07-16

**Authors:** Marili Sell, Gristin Rohula-Okunev, Priit Kupper, Ivika Ostonen

**Affiliations:** Institute of Ecology and Earth Sciences, University of Tartu, Tartu, Estonia

**Keywords:** growth strategy, belowground, exudation, respiration, pioneer root, climate change

## Abstract

Trees that are categorised by their light requirements have similarities in their growth strategies and adaptation mechanisms. We aimed to understand the complex responses of elevated air humidity on whole tree fine root carbon (C) exudation (Ex_C_) and respiration rate, morphology, and functional distribution in species with different light requirements. Three light-demanding (LD) species, *Populus × wettsteinii*, *Betula pendula*, and *Pinus sylvestris*, and two shade-tolerant species, *Picea abies* and *Tilia cordata* saplings were grown in growth chambers under moderate and elevated air relative humidity (eRH) at two different inorganic nitrogen sources with constant air temperature and light availability. The proportion of assimilated carbon released by Ex_C,_ and respiration decreased at eRH; up to about 3 and 27%, respectively. There was an indication of a trade-off between fine root released C and biomass allocation. The elevated air humidity changed the tree biomass allocation and fine root morphology, and the responses were species-specific. The specific fine root area and absorptive root proportion were positively related to canopy net photosynthesis and leaf nitrogen concentration across tree species. The variation in Ex_C_ was explained by the trees’ light-use strategy (p < 0.05), showing higher exudation rates in LD species. The LD species had a higher proportion of pioneer root tips, which related to the enhanced Ex_C_. Our findings highlight the significant role of fine root functional distribution and morphological adaptation in determining rhizosphere C fluxes in changing environmental conditions such as the predicted increase of air humidity in higher latitudes.

## 1 Introduction

Global warming promotes geographical variability in climate, whereas trends are different for lower and higher latitudes of the Northern Hemisphere (Diffenbaugh and Field, 2013; IPCC, 2021). The air temperature increase in northern latitudes is accompanied by precipitation, which is predicted to increase by 10–20% by the end of this century (Jaagus and Mändla, 2014; Kjellström et al., 2018). The higher frequency of rainfall is tightly coupled with foliage wetting in tree canopies and increases air relative humidity (RH) because of the evaporation of intercepted water (Von Arx et al., 2012; Betts et al., 2014). Moreover, RH in a warm season has risen to 10% per decade in northern Europe, Asia, and some regions of Canada from 1979 to 2014 (Vicente-Serrano et al., 2018). Recent findings indicate that RH is a more important driver of terrestrial evapotranspiration than temperature (Xiao et al., 2020).

Elevated RH combined with high soil water content may have a negative effect on leaf transpiration rate, net photosynthesis, and nutrient content (Tullus et al., 2012a; Sellin et al., 2013, 2017). Decreased transpiration rate can diminish nutrient uptake from the rhizosphere and further accrete the retardation of tree growth (Sellin et al., 2013, 2017, Cramer et al., 2009). Several studies focus on the effects of elevated RH on trees’ aboveground physiology and growth, yet only few studies show the changes in belowground. Parts et al. (2013) and Rosenvald et al. (2014) have found that silver birch trees acclimatize to elevated air humidity by allocating more carbon to fine root biomass and increasing the number of root tips to provide a sufficient level of nutrient acquisition. The acclimation range seems to be species-specific, while silver birch had a better acclimation capacity to higher air humidity than hybrid aspen (Sellin et al., 2017). Studies about the effects of elevated RH on the above- and belowground physiology of coniferous tree species, which dominate the northern latitudes, are lacking. Research concerning young trees with different light strategy could provide us crucial insights into forest regeneration in future climate scenarios.

Tree species can be categorised into two groups by their light requirements: light demanding (LD), those which emerge first after disturbances and cope well with high light conditions, and shade tolerant (ST), those which thrive in shaded conditions, especially at the beginning of growth and start to dominate at the later phase of the stand (Strauss-Debenedetti and Bazzaz, 1991; dos Santos et al., 2019). Selaya and Anten (2010) have shown that both early and late successional species can achieve similar lifetime carbon gain per unit leaf mass in natural secondary forests. Whereas the shade-tolerant late-successional species have specific life history adaptations such as producing shade leaves with high specific absorbance in low light conditions to achieve the net carbon gain similar to the light leaves (Hagemeier and Leuschner, 2019). Compared to shade-tolerant species, the light-demanding species usually have a higher net photosynthesis rate and leaf nutrient content per unit leaf area (Bazzaz, 1979; Walters and Reich, 1996; Reich et al., 1998). Although there might be a photosynthesis rate driven effect on belowground physiology, including C flux into the rhizosphere, little is known about the relationships between aboveground and belowground physiology and functioning under elevated air humidity conditions.

The most limiting mineral nutrient for plant growth is nitrogen (N), which is taken up by the roots in organic (such as amino acids and urea) or inorganic forms (nitrate, NO_3_^-^ or ammonium, NH_4_^+^) (Miller and Cramer, 2005; Bhatla and Lal, 2018). The plants’ uptake preference for a particular nitrogen ion in soil depends on different physiological and environmental factors (Britto and Kronzucker, 2013). Ammonium is more efficiently used in the synthesis of essential organic compounds and reducing NO_3_^-^ to NH_4_^+^ inside plant requires a considerable amount of fixed carbon (Bhatla and Lal, 2018). In order to increase the accumulation of necessary nutrients and water, the LD species have a fine root system that expands fast into new areas in soil with higher specific root length (SRL) and low root tissue density (RTD) compared to slow-growing shade-tolerant species (Reich et al., 1998; Comas et al., 2002). Fast-growing LD species have also shown to increase the volume of soil explored with smaller root diameters and more root tips (Comas and Eissenstat, 2004).

Fine roots are short-living and phenotypically plastic and are expected to reflect environmental changes by adapting their traits accordingly (Lõhmus et al., 1989; Ostonen et al., 2007). Fine roots consist of absorptive root tips with primary structure and nutrient-transporting roots which are characterized by secondary growth. In addition, woody roots form anatomically and functionally different primary-growth root tips, characterised by heterorhizy (Kubikova, 1967; Sutton and Tinus, 1983). Absorptive root tips are exploitative and active in the uptake of water and nutrients and are mostly mycorrhizal (Guo et al., 2008; Ostonen et al., 2017), whereas pioneer root tips are exploratory and spreading the root system and becoming transport roots in their basal part (Sutton and Tinus, 1983; Bagniewska-Zadworna et al., 2012; Freschet et al., 2021). Plant fine roots exude primary or secondary metabolites either passively or actively and with those exudates, plants can change soil chemical or physical conditions, mediate nutrient availability, or influence rhizosphere microbial communities (Jones et al., 2004; Haichar et al., 2014). The composition and rate of exudates change in response to environmental stimuli, many studies show an increase in root exudation rate in drought or nutrient deficiency conditions (Preece et al., 2018; Meier et al., 2020) and microbial activity is higher near plant root tips (Ostonen et al., 2017). Fine root C exudation rate can be predicted by fine root morphological traits, such as root tissue density and SRL (Sun et al., 2021), whereas the fine root functional proportions are highly important in understanding fine root traits and functioning. Fast-growing species have higher fine root respiration compared to slow-growing species (Comas et al., 2002), which indicates active root maintenance and new root growth (Lambers et al., 2008).

We studied three light-demanding tree species *Populus × wettsteinii*, *Betula pendula*, *Pinus sylvestris*, and two shade-tolerant species *Picea abies* and *Tilia cordata*. Hybrid aspen (*Populus×wettsteinii* Hämet-Ahti) is a fast-growing deciduous tree species favoured in short-rotation plantations in Northern Europe (Tullus et al., 2012b). Silver birch (*Betula pendula* Roth.) is a deciduous pioneer tree species widely distributed in boreal climate zone. Scots pine *(Pinus sylvestris* L.) is one of the northern hemisphere’s most widely distributed coniferous tree species because of its wide ecological amplitude (Hytteborn et al., 2005). Both silver birch and Scots pine are the dominant tree species in terms of growth stock and forest area in Estonia, covering 29% and 31% of the total forest land area, respectively (Estonian Environment Agency, 2022). Norway spruce (*Picea abies* (L.) H. Karst.) is a widely distributed species in Eurasia and, with its shallow root system, rather vulnerable to changing topsoil conditions (Seppä et al., 2009). The small-leaved linden (*Tilia cordata* Mill.) can grow vigorously under a closed overstorey (Radoglou et al., 2009). With climate warming, the growth area of *T. cordata* has been predicted to be distributed from the current most northern area in south Finland even further north (Hemery et al., 2010; Eaton et al., 2016).

This study aimed to investigate the fine root carbon exudation rates and related morphological and functional fine root traits in shade-tolerant and light-demanding tree species at moderate and elevated air humidity growing on soils with different initial inorganic nitrogen sources (NO_3_^–^ and NH_4_^+^). Furthermore, there is a limited understanding of rhizosphere C fluxes and their potential dependence on tree species’ light demand and trees’ physiological characteristics such as photosynthesis rate, transpiration, and leaf nutrient concentrations. Finding patterns in rhizosphere C fluxes, such as fine root carbon exudation and respiration, as well as fine root morphology and functional distribution for functionally similar groups of trees would allow us to improve predictions of the environmental change effects on forest soil C fluxes and adapt forest management in the future. A conceptual framework of our study is shown in the **Supplementary Data** (**Supplementary Figure 1**).

We hypothesised that: 1) to meet the increased need for mineral nutrients, light-demanding species exhibit a higher rate of carbon exudation, allocating freshly assimilated carbon belowground; 2) the elevated air humidity increases the fine root exudation and respiration rates, which is mediated by the changed soil nitrogen source, changes in fine roots morphology and shifts in functional distribution; 3) increase in fine root exudation and respiration declines biomass allocation of tree saplings.

## 2 Methods

### 2.1 Experimental design

Altogether 20 *Populus × wettsteinii* (one-year-old), 20 *Betula pendula* (two-year-old), 32 *Pinus sylvestris* (five-year-old), 24 *Picea abies* (three-year-old), and 12 *Tilia cordata* (four-year-old) saplings were grown in Percival AR-95 HIL (Percival Scientific Inc. USA) growth chambers. Throughout the experiments, the growth chambers were held at constant temperature (∼21.6 °C) and light intensity (∼600–800 μmol m^−2^s^−1^ at the top of the saplings). The length of night and day in the chambers were 8 h and 16 h, respectively. The air humidity treatment consisted of moderate (mRH), where chamber air relative humidity (RH) values were set at 80% and 65% during night and day, respectively, and elevated (eRH), where the chamber RH values were set at 80% during both night and day. The chamber characteristics (air flow, spectrum of the lamps/light source) were identical between mRH and eRH chambers. To ensure independent verification of the set values of the air humidity and temperature in the chambers, the chambers were provided with additional air temperature and RH sensors (HMP45A, Vaisala) and data was recorded with a DL2e data logger (Delta-T Devices). The daily variations of air temperature and RH between chambers is shown in **Supplementary Data** (**Supplementary Figure 2**). Each tree sapling was growing in a separate 10 L box filled with sphagnum peat mixed with gravel and milled limestone. The growth substrate for the trees grown under nitrate treatment contained 1.12 g NO_3_^−^ -N, 0.1 g NH_4_^+^-N, and 1.45 g Ca (calcium ammonium nitrate), whereas the growth substrate of the trees with the ammonium treatment contained 1.10 g NH_4_^+^-N, 0.1 g NO_3_^−^ -N (ammonium sulphate and ammonium nitrate), and 1.14 g S (ammonium sulphate). Additionally, the mineral elements added to the growth substrate per box were 0.33 g P (P_2_O5), 1.24 g K (K_2_O), 0.27 g Ca (CaO), 0.27 g Mg (MgO, MgOSO_3_), and 0.56 g S (SO_3_, MgOSO_3_). The saplings were fertilised with 2 mL Agrimix-Micro Profi (INTERMAG, Poland), to provide microelements (B, Cu, Fe, Mn, Mo, Zn). The trees were weighed and watered every day to restore the soil water reserve and to maintain the upper limit of the soil water content at 60% of the field capacity. The soil surface of the pots was covered with aluminium foil, to prevent soil evaporation. From the pot weighting data, the daily water loss (g) of each sapling was calculated. To calculate the whole tree transpiration rate at the end of the study, the average water loss was expressed per unit leaf area (g m^-2^ h^-1^).

### 2.2 Measurements

The fine root carbon exudates were collected based on the culture-based cuvette method (Phillips et al., 2008) at the end of the experiment. Each sapling was removed from the growth box and the soil surrounding the roots was wrapped in cling film. After cutting a window into the cling film, an intact root was selected for measurement of root exudation and later analysis. The saplings of *Picea abies* could not be removed from the growth box without damaging the roots because they were more brittle than other species’ roots. Therefore, for *P. abies*, the box was placed on its side, and a suitable fine root segment was washed out with flowing tap water, cleaned carefully, and incubated inside the growth box. The chosen root segment was placed inside a cuvette (30 or 50 mL) which was filled with ∼20 mL of sterile glass beads (∅ 0.5–1.25 mm) to imitate mechanical support and soil porosity for the root segment. The cuvette was sealed with rubber cork covered with parafilm with a wedge cut for the root. The cuvette was then filled with carbon-free solution (0.5 mM NH_4_NO_3_, 0.1 mM KH_2_PO_4_, 0.2 mM K_2_SO_4_, 0.2 mM MgSO_4_, and 0.3 mM CaCl_2_), and the cuvette was sealed with parafilm. After 24h of stabilisation, the root segments were flushed three times with a clean carbon-free solution and incubated for 24h with a fresh solution. The exudated solution was collected, and total organic carbon (TOC, mg C l^−1^) was determined by a TOC analyser (Elementar GmbH, Germany). The total fine root dry weight was measured to calculate the fine root carbon exudation per tree (Ex_C_, mg C day^−1^).

The WinRHIZO™ Pro (Regent Instruments Inc. 2003) was used to measure sample root length (cm), root surface area (cm^2^), average root diameter (AD, mm), root volume (cm^3^), and the number of root tips. From these fine root characteristics, different parameters were calculated: the specific root area (SRA = surface area/mass, m^2^ kg^−1^), specific root length (SRL = length/mass, m g^−1^), root tissue density (RTD = mass/volume, kg m^−3^), and branching intensity (BI = tips/mass, mg^−1^) (Ostonen et al., 1999). In addition, the exudation root samples were separated according to their primary function into absorptive (first and second-order roots), pioneer (long root tips with primary structure as early-stage transport roots), and transport roots (long woody roots) and measured separately with WinRHIZO™ Pro. Absorptive (absorb), pioneer (pioneer), and transport (transp) root proportion (%) of total fine root dry weight (DW) were calculated. All (sub)samples were dried at 65°C for 48h and weighed.

Fine root respiration was measured on root samples separate from exudation root samples with a CIRAS-2 portable photosynthesis system (PP Systems, Amesbury, MA, USA) equipped with a conifer cuvette. The chosen root sample was cleaned from soil particles, and the respiration values were recorded after excision ~5 min when the readings were stabilised. The parameters of the cuvette were: temperature 22°C, external CO_2_ concentration ~400 ppm, and humidity >90% to avoid drying the root sample during measurement. Total fine root dry weight was measured to calculate the whole tree fine root respiration rate (R-DW, μmol CO_2_ s^−1^).

The net photosynthesis rate (P_n_) of deciduous leaves was measured with a CIRAS-2 leaf cuvette at constant irradiance (800 μmol m^−2^ s^−1^). *P. wettsteinii* and *T. cordata* sample leaves were randomly selected from the upper canopy and in *B. pendula*, similarly from the top of the upper canopy, but the third, fifth, and seventh leaves were measured for photosynthesis. In the case of *P. sylvestris* and *P. abies*, the conifer cuvette was used to measure the P_n_ of shoots. In *P. sylvestris* one previous and two current year shoots were selected and for *P. abies* two current-year shoots were selected for measurement (each bearing ~60 needles). The daytime gas exchange was measured at PAR of 400 μmol m^−2^ s^−1^ using the external light unit of the cuvette. The gas exchange measurements were conducted at constant temperature (22°C), external CO_2_ concentration (~400 ppm), and ambient RH (≤70% inside the cuvette). The leaf area of deciduous trees was measured with LI-3100C (LI-COR Biosciences, USA) at the end of the experiment. For coniferous species, a subsample of 100 needles projection area was measured with WinRHIZO™ Pro. The total needle area was calculated based on the sample needle area and dry mass, and the total needle dry mass. The biomass of each tree part (leaves/needles, stem, coarse roots, fine roots) was dried separately at 65 °C for at least 48 h and the dry mass was weighed. The N concentration of leaves and needles was determined using a Kjeltec Auto 1030 analyser (FOSS Tecator AB, Höganäs, Sweden); the P and K concentrations were determined spectrophotometrically from the Kjeldahl digest using a FIAstar 5000 analyser (FOSS Tecator AB).

### 2.3 Statistical analysis

Statistical analysis was conducted in Statistica 7.1 (StatSoft Inc. USA), and RStudio Version 1.3.1093 (RStudio, PBC, MA, USA), whereas some figures were made by using Microsoft Office Professional Plus 2019 version 1808. First, we used the factorial ANOVA to determine any effects of tree species, air relative humidity or nitrogen source on the canopy net photosynthesis, transpiration rate, whole tree fine root carbon exudation rate and whole tree fine root respiration rate (**Supplementary Data**, **Supplementary Table 1**). While the soil nitrogen source did not affect any of the mentioned parameters, the data of both N treatments were pooled together for further analysis. Tukey HSD test was used to determine the effects of elevated air humidity on the forementioned parameters. Non-parametrical tests were used when data was not normally distributed. The ratios of whole tree fine root exudation and respiration to canopy net photosynthesis were analysed with non-parametrical Kruskal-Wallis ANOVA test. Both parametrical (Pearson) and a non-parametrical correlation (Spearman) was used to analyse the relationship between fine root exudation and respiration rate.

Tukey HSD test was used to determine the effects of elevated air humidity on fine root morphological traits, leaf nutrient concentrations and biomass within and between tree light-use strategy groups. The coefficient of variation (cv = 100×(SD/mean)) was checked for absorptive, pioneer, and transport root proportions (dry weight, %) for all tree species (Ostonen et al., 2007). The correlations between fine root proportions and fine root morphological traits were determined.

Redundancy analysis (RDA, Canoco, ter Braak and Šmilauer, 2002) was used to explain the variation of fine root morphological traits (AD, SRA, SRL, RTD, BI), including fine root carbon exudation rates (per dry mass) by following datasets 1) plant physiological variables and 2) proportions of functionally different roots within fine roots as explanatory variables. The plant physiological variables were net photosynthesis rate, transpiration rate, leaf to fine root dry mass ratio, leaf N, P, K concentration, and fine root respiration rate. The functional distribution indicating variables were absorptive, pioneer, and transport root proportions (DW%) within the fine root sample. To assess the effect of each explanatory dataset on each tree species partial canonical analysis (forward selection) was performed at P < 0.05 level. The significance of the RDA results was tested with a permutation test (Monte Carlo test (999); P < 0.01).

## 3 Results

### 3.1 Trees aboveground and belowground traits

**Figure 1:**
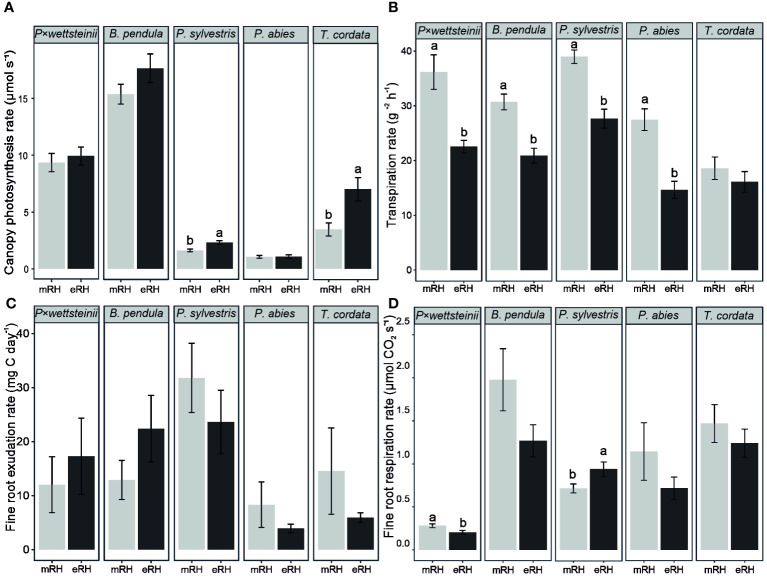
Average ± standard error of whole canopy net photosynthesis rate **(A)**; transpiration rate **(B)**; fine root carbon exudation per tree root system **(C)**, and fine root respiration per tree root system **(D)** of *Populus × wettsteinii, Betula pendula, Pinus sylvestris, Picea abies*, and *Tilia cordata.* The letters indicate the significant differences between moderate (mRH) and elevated air relative humidity (eRH) treatments within one species (P < 0.05). The data of *P. × wettsteinii, B. pendula*, and *P. sylvestris* are from Sell et al. (2022).

The canopy net photosynthesis rate was, on average, 9.7 ± 0.5, 16.5 ± 0.7, and 5.2 ± 0.7 μmol s^−1^ for *P. × wettsteinii*, *B. pendula* and *T. cordata*, respectively, and 2.0 ± 0.1 and 1.1 ± 0.1 μmol s^−1^ for *P. sylvestris* and *P. abies*, respectively (**Figure 1A**). Among all tree species, *P. sylvestris* and *T. cordata* showed an increased canopy photosynthesis rate at eRH compared to mRH (P < 0.05). The average transpiration rate of *P. × wettsteinii*, *B. pendula* and *T. cordata* was 29 ± 2, 26 ± 1 and 17 ± 1 g m^−2^ h^−1^, respectively (**Figure 1A**). The average transpiration rate of *P. sylvestris* and *P. abies* were 33 ± 1 and 21 ± 2 g m^−2^ h^−1^, respectively (**Figure 1B**). The transpiration rate decreased in all species at elevated air humidity treatment (P < 0.05), except for *T. cordata*.

There was no significant effect of elevated air humidity treatment on the whole tree C exudation rates within tree species (**Figure 1C**). The average C exudation rate per whole tree fine root DW of light-demanding species (*P. × wettsteinii*, *B. pendula*, and *P. sylvestris*) was 21.7 ± 2.5 mg C day^-^¹, whereas the C exudation rate per whole tree fine root DW of shade-tolerant species (*T. cordata* and *P. abies*) was, on average, 7.3 ± 1.8 mg C day^-^¹ (**Figure 1C**). The light-demanding tree species had significantly higher average fine root carbon exudation rate per whole tree fine root dry weight than the shade-tolerant tree species (P < 0.05), irrespective of the humidity treatment.

The whole tree fine root respiration was affected by the elevated humidity in *P. × wettsteinii* and *P. sylvestris* (**Figure 1D**). *P. × wettsteinii* trees growing on eRH had lower whole tree fine root respiration rates, compared to control mRH conditions (P < 0.05). *P. sylvestris* which were growing on eRH had a higher whole tree fine root respiration rate than growing under mRH. The fine root C exudation correlated positively with the fine root respiration rate only in *P. abies* (R^2^ = 0.45, P < 0.05).

The proportion of exuded carbon of assimilated carbon from whole tree net photosynthesis (Ex_C_/P_n_) was highest in *P. sylvestris*, about 5% (**Table 1**). The Ex_C_/P_n_ decreased in *P. sylvestris* and *T. cordata* at elevated air humidity compared to control conditions. The Ex_C_/P_n_ was lowest in *P. wettsteinii*, and *B. pendula*, about 0.3%, with no difference between air humidity treatments. The proportion of carbon respired of assimilated carbon from whole tree net photosynthesis (R_DW_/P_n_) was highest in *P. abies*, about 90% (**Table 1**). At elevated air humidity, the R_DW_/P_n_ decreased in deciduous species *P. wettsteinii*, *B. pendula*, and *T. cordata* (P < 0.05).

**Table 1:** The proportion (%, standard error in brackets) of fine root exudation (ExC, μmol C/day) and respiration (RDW, μmol C/day) of assimilated carbon from whole tree net photosynthesis (Pn, μmol C/day) across five tree species.

	*Populus ×wettsteinii*		*Betula* *pendula*		*Pinus sylvestris*		*Picea* *abies*		*Tilia* *cordata*	
	mRH	eRH	mRH	eRH	mRH	eRH	mRH	eRH	mRH	eRH
Ex_C_/P_n_	0.3 (0.1)	0.4 (0.1)	0.3 (0.1)	0.4 (0.1)	6 (1)a	3 (1)b	2 (1)	1.5 (0.5)	1.3 (0.5)a	0.3 (0.1)b
R_DW_/P_n_	3 (0.2)a	2 (0.2)b	13 (3)a	7 (1)b	46 (3)	40 (3)	107 (28)	78 (17)	46 (6)a	19 (3)b

The letters indicate a significant difference between moderate (mRH) and elevated air humidity (eRH) treatments within one species (P < 0.05).

**Figure 2:**
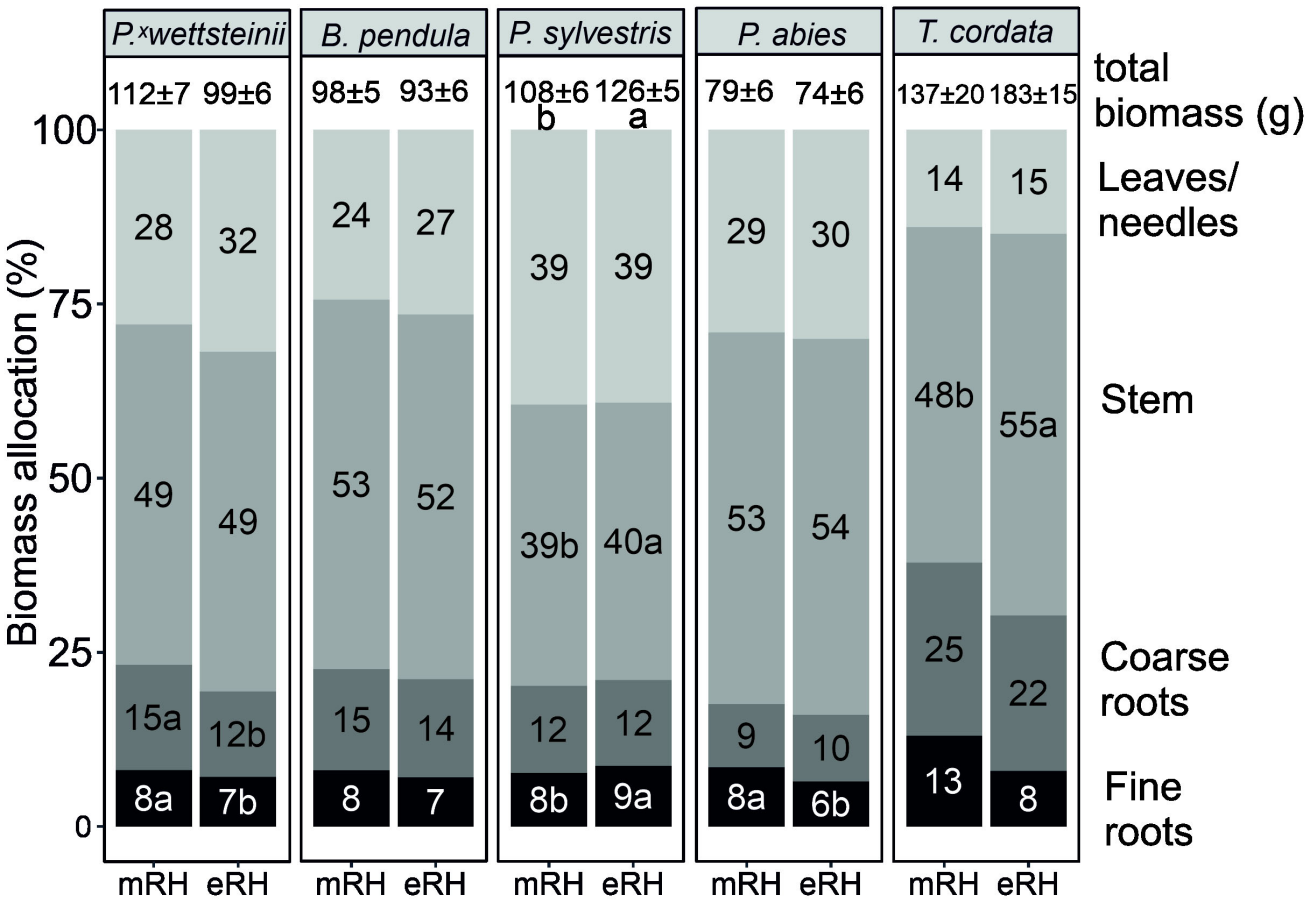
Biomass (leaves/neeldes, stem+stump, coarse roots, and fine roots) allocation (% of total) and total biomass (g) of *Populus × wettsteinii, Betula pendula, Pinus sylvestris, Picea abies*, and *Tilia cordata* at either moderate (mRH) or elevated (eRH) humidity treatments. The letters indicate the difference in biomass distribution (absolute values) within one species between air humidity treatments (P < 0.05).

The foliar N, P, and K concentrations in leaves/needles were highest in LD species, 2.48, 0.21, and 0.98, respectively, compared to ST species, 2.04, 0.16, and 0.72, respectively. The average total biomass and the biomass allocations of *P. × wettsteinii*, *B. pendula*, *P. sylvestris*, *P. abies*, and *T. cordata* at different air humidity treatments are presented in **Figure 2**. The total biomass increased at eRH only in *P. sylvestris*, compared to control conditions (P < 0.05). The foliar biomass was significantly higher in LD species, 35.9 ± 1.4 g compared to ST species, 22.8 ± 1.2 g. The stem biomass was significantly higher under elevated air humidity for *P. sylvestris* and *T. cordata*. The fine root biomass for *P. sylvestris* was higher under elevated air humidity but for *P. abies* and *P. wettsteinii* the fine root biomass was lower under eRH compared to mRH (P < 0.05).

### 3.2 Fine root traits

The specific fine root area for *B. pendula* increased at eRH, while RTD decreased. The AD increased at eRH for *P. sylvestris*, while SRA, SRL, and BI decreased, compared to fine roots in mRH (Sell et al., 2022). *P. abies* had lower RTD in eRH, compared to mRH (**Supplementary Data**, **Supplementary Table 2**). There was no significant difference in fine root traits of *P. × wettsteinii* or *Tilia cordata* in response to air humidity treatment.

**Figure 3:**
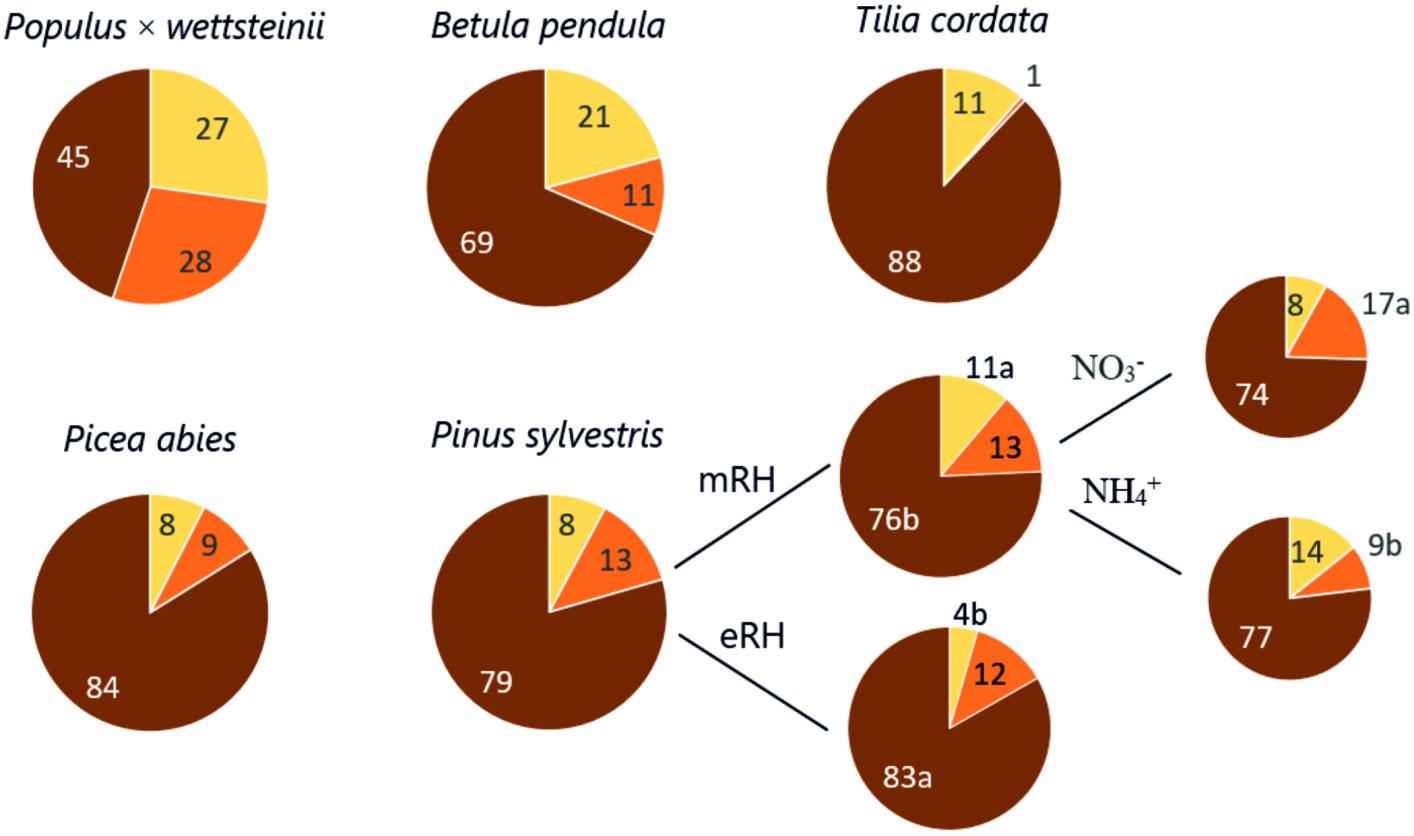
The pie chart shows the proportion of absorptive (yellow), pioneer (orange), and transport (brown) root dry weight within the fine root sample *Populus* × *wettsteinii*, *Betula pendula, Pinus Sylvestris* (Sell et al., 2022), *Picea abies, Tilia cordata*. Moderate (mRH) and elevated air relative humidity (eRH) conditions, and soil nitrogen sources (NO_3_^-^ - nitrate, NH_4_^+^ - ammonium, separately shown for *P. sylvestris*.

The fine root functional distribution into absorptive, pioneer, and transport roots of each tree species is shown in **Figure 3**. The variation in absorptive root DW% of fine roots DW was highest in *P. × wettsteinii* followed by *P. sylvestris* and *P. abies* (70–100%), lowest for *B. pendula* and *T. cordata* (~50%). The highest variation in pioneer root DW% of fine roots was for *B. pendula* and *T. cordata* (102–108%), lowest for conifer species (50%). Higher air humidity decreased the absorptive and transport root proportions in *P. sylvestris* and at moderate air humidity, the proportion of pioneer roots was higher in the NO_3_^-^ source, compared to the NH_4_^+^ treatment (Sell et al., 2022). The N source and RH treatment did not affect other species’ fine root proportions (**Figure 3**).

**Figure 4:**
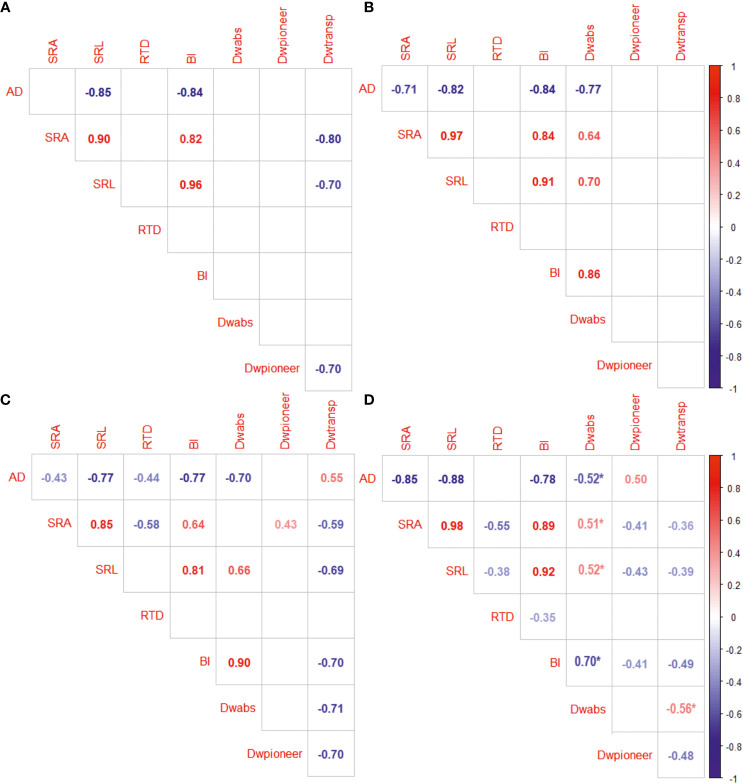
The correlation between the proportions (%) of absorptive (abs), pioneer, transport (transp) roots of fine roots (on dry weight (DW) basis) and fine root morphological traits (AD, average diameter, mm; SRA, specific root area, m^2^ kg^−1^; SRL, specific root length, m g^−1^; RTD, root tissue density, kg m^−3^; BI, branching intensity, mg^−1^) of *Betula pendula* **(A)** *Tilia cordata* **(B)**, *Pinus* sylvestris **(C)**, and *Picea abies* **(D)**. *Populus* × *wettsteinii* had no significant correlations. Only significant correlations are presented. Asterix indicates Pearson correlation.

The values of fine root morphological traits depend on fine root functional proportioning, which, however, is dependent on the tree species (**Figure 4**). Absorptive root proportion (%) of fine roots was negatively correlated with the average fine root diameter in *P. sylvestris* and positively with SRA, SRL, and BI in most species. The proportion of pioneer root of fine root DW was positively correlated with AD and negatively correlated with SRA, SRL, and BI in *P. sylvestris*. The share of transport roots correlated positively with fine root AD but negatively with SRA, SRL, and BI. None of the fine root functional group proportions correlated with fine root RTD.

### 3.3 Combined analysis

About 43% of the root morphological variation was explained by the difference between deciduous and coniferous species, including by the net photosynthesis rate, N and K concentration in leaves, and functional distribution of fine roots (**Figure 4**). Higher fine root SRA, SRL, and BI along the first axis were related to the higher photosynthesis and nitrogen content in leaves, that was mostly driven by *P. × wettsteinii* and *B. pendula*. Furthermore, the increase of SRA correlated positively with the proportion of absorptive roots within fine roots. Average fine root diameter increased towards coniferous tree species. The light-demanding species were located along the second axis, which explained approximately 15% of the variation in fine root traits and correlated positively with fine root RTD and negatively with the C exudation rate. Fine root C exudation rate increased towards light-demanding tree species and was related to transpiration rate and a higher proportion of pioneer roots within fine roots. An increase in fine root RTD towards shade-tolerant tree species correlated with the higher share of transport roots and the P concentration in leaves.

## 4 Discussion

### 4.1 Trees’ nutrient demand under elevated air humidity

The light-demanding species showed higher whole tree net photosynthesis rate compared to shade-tolerant species which has shown before in earlier studies (Bazzaz, 1979; Walters and Reich, 1996; Reich et al., 1998). Unexpectedly, the elevated RH increased the P_n_ in *P. sylvestris* and *T. cordata*. (**Figure 1A**). Lower water pressure deficit gradient between leaf and surrounding air at eRH supports stomatal opening and therefore facilitates carbon assimilation via photosynthesis (Oksanen et al., 2019). The average leaf nutrient concentrations (nitrogen, phosphorous, potassium) of each tree species was in the optimum range (according to data comparison with Ingestad, 1962; Pigott, 1991; Tullus et al., 2007), proving the availability of necessary nutrients. The initial soil nitrogen source did not affect the canopy photosynthesis nor the transpiration rate; however, most of the studied tree species had a lower transpiration rate at elevated RH compared to control conditions (**Figure 1B**). Due to the decreased transpiration rate, the mass flow of water and soluble nutrients reaching fine roots in soil may decrease (Cramer et al., 2009) and therefore initiate belowground physiological processes for compensation.

**Figure 5:**
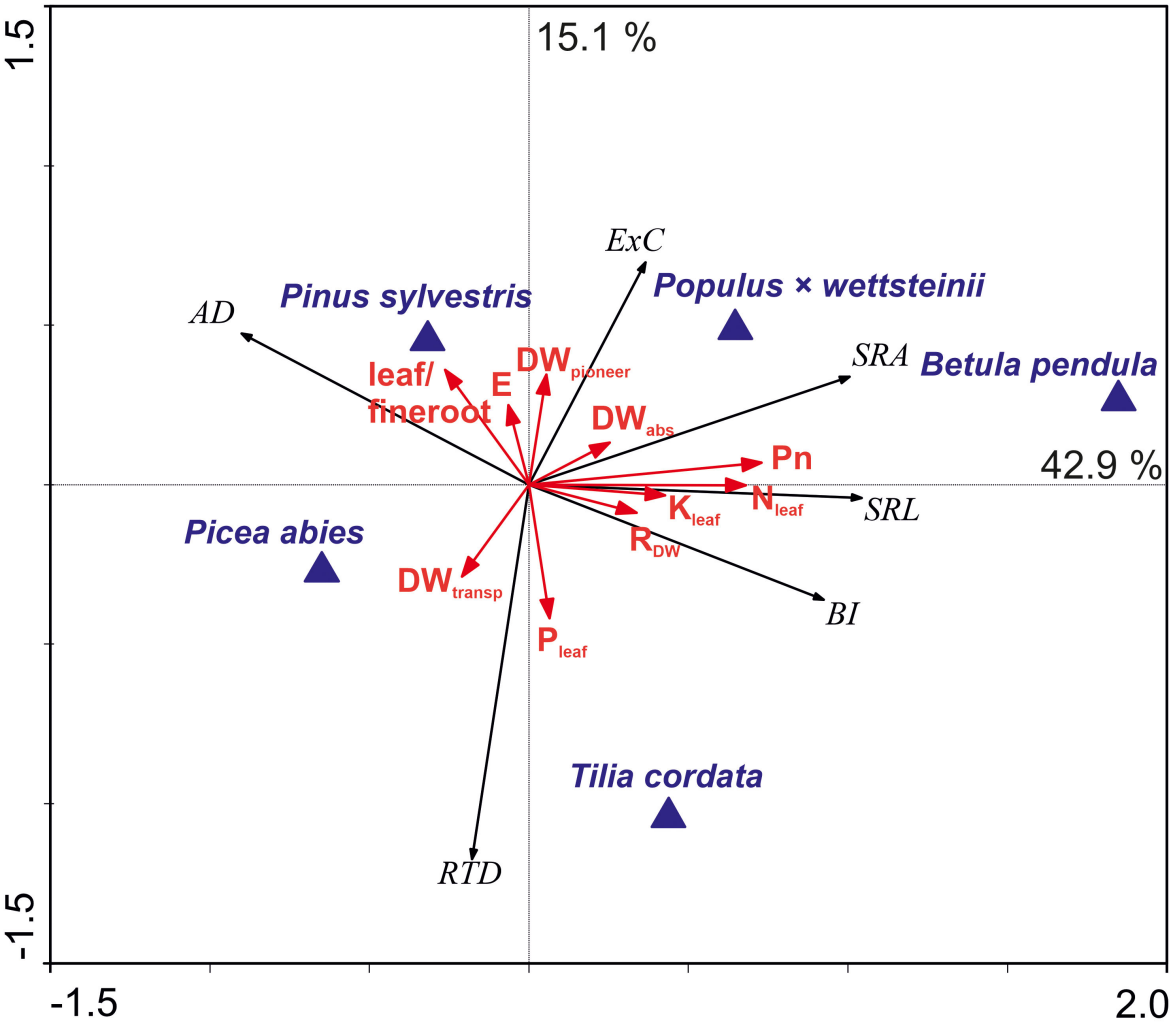
The ordination biplot based on redundancy analysis (RDA) of the fine root morphological traits and carbon exudation rates in relation to the proportion per dry weight (DW%) of the absorptive, pioneer, and transport roots of the fine root sample, R-DW – fine root respiration per whole tree fine root DW (μmol CO_2_ s^-1^), P_n_ – canopy photosynthesis rate (µmol s^-1^), E – transpiration rate (g m^-2^ h^-1^), leaf nitrogen (N) potassium (K), phosphorous (P) concentration, and leaf/fine root ratio (red arrows) and the tree species (blue triangles). The relative eigenvalues of axis one and axis two were 42.9% and 15.1%, respectively. The model described 64.8% of the variation in fine root traits (999 Monte Carlo permutation test, P = 0.001). Abbreviations for dependent variables: AD, mean fine root diameter (mm); SRA, specific root area (m^2^ kg^−1^); SRL, specific root length (m g^−1^); RTD, root tissue density (kg m^−3^); BI, branching intensity (mg^−1^); Ex_C_, whole tree fine root carbon exudation (mg C day^−^¹).

Plants mediate nutrient availability in soil by releasing different metabolites, and the fine root exudation rate could depend on the plant nutrient demand (Phillips et al., 2011). The rapid release of exudates has been expressed in species with an acquisitive strategy to enhance plant N uptake (Guyonnet et al., 2018; Sun et al., 2021). Low P availability may initiate a higher fine root exudation rate (Jiang et al., 2022) which supports the fact that lower P concentration in leaves promotes a higher fine root exudation rate (**Figure 5**). The increase in air humidity did not change the total fine root C exudation rates of five different tree species, nor did the initial dominant nitrogen forms (NO_3_^-^, NH_4_^+^) in the soil. Therefore, we assume that the nutrient availability was not limited for all the studied tree species, and the change in fine root morphology and functional distribution compensated the effect of environmental change on the C exudation rate at the tree level.

### 4.2 Trees’ acclimation under elevated air humidity

The increase in fine root biomass at elevated RH was suggested as an acclimation mechanism for roots to increase the acquisition of water and nutrients (Rosenvald et al., 2014). The change in biomass allocation under elevated air humidity was not consistent over different tree species (**Figure 2**). However, the tree species with increased stem biomass showed a tendency of decreased fine root C exudation rate at eRH, and the proportion of exudation to assimilated carbon via photosynthesis was significantly lower in more humid conditions (**Table 1**). Considering the carbon-nutrients trade-off theory (Franklin et al., 2012), and that *P. sylvestris* and *T. cordata* in this study were not limited by mineral nutrients under elevated air humidity, the assimilated carbon was rather used for growth than for improving nutrient acquisition.

Fine root exudation rate has been shown to correlate positively with fine root respiration rate (Sun et al., 2021), which in our study applied only for *P. abies*. The respiration rates were not distinctively different between phylogenetically different species (**Figure 1D**), although studies have suggested that deciduous species have higher photosynthetic capacity and higher N content per leaf compared to coniferous species, which also advance the higher respiration rate in deciduous forests (Reich et al., 1998; Raich and Tufekciogul, 2000; Takashima et al., 2004; Sun et al., 2017, 2021; Han and Zhu, 2020). A field study with *B. pendula* showed decreased soil respiration at elevated air humidity, as acclimation of long-time changes in atmospheric humidity by regulating carbon-cycle processes (Kukumägi et al., 2014), similarly *P. ×* wettsteinii and *B. pendula* had proportionally less photosynthetic carbon respired via roots at elevated RH (**Table 1**). *P. sylvestris*, however, had an opposite reaction, increased root respiration at eRH, which was likely driven by the fresh assimilates from increased photosynthesis, seen similarly by Ekblad and Högberg (2002). Autotrophic respiration is divided into growth respiration, including the carbon used for the production of new tissues, and maintenance respiration to maintain the new tissues (Lambers et al., 2002), which fits with the increased proportion of fine roots in *P. sylvestris* at eRH (**Figure 2**). However, since the effect of elevated air humidity on fine root respiration was species-specific, further studies based on tree functional groups are needed.

### 4.3 Fine root morphology and functional distribution

Fine root morphological traits variation seems not to be explained by the elevated air humidity, nor the initial soil nitrogen source, but by the phylogenetic origin, while the distances observed between coniferous species were smaller compared to the distances between coniferous and deciduous trees (**Figure 5**). The variation in traits among phylogenetically distinct groups can be attributed to the species’ evolutionary history and their adaptation to natural habitats. Evolutionally older taxa have, on average, thicker roots and are more dependent on mycorrhizal symbiosis to forage nutrients than younger taxa (Ma et al., 2018). In our study, the coniferous tree species’ fine roots were thicker (**Supplementary Data**, **Supplementary Table 2**). Deciduous species fine roots SRA and SRL were higher compared to the studied coniferous species (**Supplementary Data**, **Supplementary Table 2**). Comas et al. (2012) suggest that some fast-growing species with high plasticity are better adapted to nutrient-poor conditions, while thinner roots with longer SRL facilitate nutrient foraging for a fast-growth strategy. In our study, differences occurred even within phylogenetically related groups, such as light-demanding *B. pendula* having higher SRA and SRL than shade-tolerant tree species *T. cordata* (**Supplementary Data**, **Supplementary Table 2**). *Betula* species can grow in nutrient-poor soils, while thin and long fine roots enhance nutrient acquisition, whereas *T. cordata* trees prefer nutrient-rich soils (Radoglou et al., 2009; Hynynen et al., 2010). *T. cordata* had higher RTD than *B. pendula*, and within coniferous species also *P. abies* (ST) had higher RTD compared to *P. sylvestris* (LD) (**Supplementary Data**, **Supplementary Table 2**). Zangaro et al. (2016) showed that early-successional light-demanding woody species were positively associated with higher SRL, whereas late successional shade-tolerant species with RTD. Higher RTD characterises tissues with high dry mass content, which might slow down the flow of metabolites and nutrients, thus, root tissue density is often considered to reflect root functioning (Ostonen et al., 2013; Freschet et al., 2021).

High SRA and SRL of fine roots were positively correlated with the share of absorptive roots within fine roots (**Figure 4**). These traits show that plant roots explore a greater volume of soil with increased contact and are associated with the most active water and nutrient uptake by absorptive root tips (Ostonen et al., 1999; Sun et al., 2021). A study by Ostonen et al. (2011) showed the share of absorptive roots within fine roots of *Picea abies* to increase in higher latitudes to adapt to climate conditions. Deciduous species had on average higher proportion of absorptive roots compared to coniferous species (**Figure 3**). Interestingly, the variation of absorptive root proportion was higher in coniferous species, compared to *B. pendula* and *T. cordata*. Correspondingly, we determined positive correlations between the fine root and absorptive root morphological traits in coniferous species, indicating that fine root morphological traits may also reflect functional plasticity at the fine root system level (data not shown).

The share of pioneer roots had a significant impact on AD of fine roots determining the value of SRL and SRA in conifers but had no impact on fine root AD in deciduous trees (**Figures 4**, **5**). The apical part of pioneer root tips is with primary structure, and the basal part of the pioneer root has a transition zone to secondary growth and faster secondary vascular tissue development than absorptive root tips (Bagniewska-Zadworna et al., 2012). According to Bagniewska-Zadworna et al. (2012), pioneer roots have a larger diameter of tracheary elements, facilitating water and nutrient transport. The high proportion of pioneer roots decreased the average RTD of fine roots in deciduous tree species and was in positive correlation with fine root SRA (**Figure 4**). High branching intensity characterises the distribution of roots in soil (Freschet et al., 2021). The BI increased with a higher share of absorptive roots indicating facilitated nutrient acquisition, whereas a higher proportion of pioneer and transport roots correlated with lower branching intensity (**Figure 4**). The functional distribution within fine roots has an impact on the morphological traits of fine roots, whereas both morphology and functional proportions depend on tree species. The functional distribution seems to be connected to the phylogenetical origin, rather than the light-use strategy of tree species.

### 4.4 Fine root carbon exudation and trees’ light use strategy

In our study, *P. sylvestris* had the overall highest fine root C exudation rate followed by deciduous species. Wang et al. (2021) showed higher fine root C exudation rates in deciduous species than in conifers. The fine root C exudation rate has been considered a competitive fine root functional trait that can be predicted by fine root morphological traits (Sun et al., 2021). Both the fine root carbon exudation rates and RTD are associated with trees’ light demand. Light-demanding tree species had higher fine root C exudation rate, and the fine root C exudation rate correlated negatively with RTD (**Figure 5**). Light-demanding trees had a higher share of pioneer roots compared to the shade-tolerant tree species and fine root C exudation rate depends on the share of pioneer roots within fine roots (Herron et al., 2013; Sell et al., 2022, **Figure 5**). Compared to adult trees, young tree roots form more pioneer roots and have a significant contribution to the entire root system, and similar future experiments should take the pioneer root effect into consideration. Pioneer root tips are important in spreading root systems, where also absorptive root tips start to form (Freschet et al., 2021).

The proportion of carbon assimilation allocated to total fine root exudation of *P. abies* saplings in control conditions of the current study was 2%, higher, compared to the 0.7% as seen in Brunn et al. (2022) in 70-year-old spruce forest. Young trees are metabolically more active and have fast active growth compared to older trees and therefore, we expect that these tree saplings growing in controlled conditions exhibit higher exudation rates. Brunn et al. (2022) showed that in a 70-year-old late successional deciduous species, *Fagus sylvatica* the exuded C of assimilated C was 0.5%, compared to our T. cordata, 1.5%. In *P. sylvestris* saplings, the share of exuded C of assimilated C was ~5%, while for fast-growing light-demanding deciduous tree species only about 0.3%. *P. wettsteinii* and *B. pendula* maintained continuous leaf growth throughout the entire experiment and the carbon assimilated was probably used for active growth and biomass increment, and therefore assimilated carbon was likely less directed into fine root exudation and respiration. Furthermore, initiated by air humidity changes, there was a trade-off between fine root carbon release (via exudation and respiration) and C allocation into stem biomass in *P. sylvestris* and *T. cordata*. Overall, the coniferous species had a high proportion of exuded and respired C of assimilated C. Therefore, the increase or decrease in fine root exudation and respiration affect the biomass allocation of the saplings. Mäkelä et al. (2022) showed a significant priming effect of root exudates, which led to a decrease in the carbon residence time in the soil. Thus, the response of fine root C exudation in various tree species might need to be considered in the selection of species used for afforestation in the Nordic countries.

## 5 Conclusion

Climate change such as increasing air humidity altered the fine root morphology and functional proportions, although the impact of the environmental change varied between tree species. While the modifications in fine root traits related to the fine root C exudation, the total fine root carbon exudation per tree did not change at elevated air humidity, indicating to an inherent acclimation mechanism of the tree fine root system. The carbon exudation rate of fine roots might also be related to the species’ light-use strategy. Light-demanding species had a higher share of pioneer roots within fine roots which affected the increase in fine root C exudation rate compared to shade-tolerant ones. Based on our results, we can emphasize a potential shift in C fluxes in response to increased air humidity. The proportion of C released through exudation in relation to C assimilated in photosynthesis decreased for two out of the five studied tree species, and this decrease may be related to the increase in stem biomass. This is an essential consideration for tree species selection in future forests under changing climate conditions.

## Data availability statement

The raw data supporting the conclusions of this article will be made available by the authors, without undue reservation.

## Author contributions

MS: Data curation, Formal analysis, Investigation, Validation, Visualization, Writing – original draft, Writing – review & editing. GR-O: Data curation, Investigation, Validation, Writing – review & editing. PK: Conceptualization, Data curation, Funding acquisition, Investigation, Methodology, Project administration, Resources, Supervision, Validation, Writing – review & editing. IO: Conceptualization, Data curation, Funding acquisition, Investigation, Methodology, Project administration, Resources, Supervision, Validation, Writing – review & editing.
