# Supplementary material for: Adapting to climate change: responses of fine root traits and C exudation in five tree species with different light-use strategy
Source: Front Plant Sci. 2024 Jul 16;15:1389569. doi: 10.3389/fpls.2024.1389569 (PMC11289846; doi:10.3389/fpls.2024.1389569)
Supplement: Supplementary file 1 [file DataSheet_1.docx]

**SUPPLEMENTARY DATA**


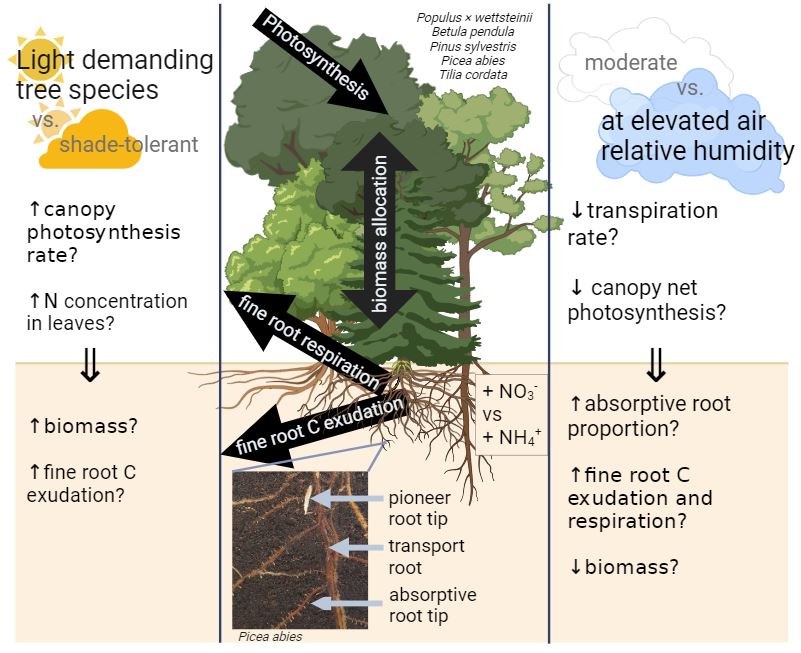


**Figure 1**. A conceptual framework of growth chamber experiments with three light-demanding tree species, *Populus × wettsteinii*, *Betula pendula*, *Pinus sylvestris*, and two shade-tolerant species *Picea abies* and *Tilia cordata*. The black arrows show the studied carbon fluxes of trees and the fine root image of *Picea abies* shows the different functional proportions (absorptive, pioneer, and transport) that we measured. On the left side, the figure shows the increased (↑) or decreased (↓) parameters we expected for light-demanding tree species (compared to shade-tolerant species). On the right side, the increased or decreased effects of elevated air relative humidity on above- and belowground parameters compared to moderate conditions. N – nitrogen; C – carbon. Image created using biorender.com site.

**Figure 2**. Example of growth chambers' daily air temperature and air relative humidity variation within four weeks (data were recorded every 10 min with HMP45A temperature and humidity sensors). The air relative humidity values for moderate conditions (mRH) were set at 80% and 65% during night and day, respectively, and elevated (eRH), where the chamber RH values were set at 80% during both night and day. Fluctuations in measured parameters are caused by day/night shifts in chambers, the opening of the chambers during watering, and measurements of plant traits.

**Table 1**. Factorial ANOVA to determine the effects of tree species, air relative humidity (RH), nitrogen (N) source and their interactions to canopy net photosynthesis rate, transpiration rate, whole tree fine root carbon exudation and respiration. ns – not significant.

|  | Canopy net photosynthesis (μmol s^−1^) | | Transpiration (g m^−2^ h^−1^) | | Fine root exudation rate (mg C dayˉ¹) | | Fine root espiration rate (μmol CO_2_ s^−1^) | |
| --- | --- | --- | --- | --- | --- | --- | --- | --- |
|  | F | P | F | P | F | P | F | P |
| Tree (species) | 218,9 | <0.001 | 23,9 | <0.001 | 3,8 | <0.01 | 13,5 | <0.001 |
| N source | 0,2 | ns | 0,7 | ns | 0,7 | ns | 0,1 | ns |
| RH | 12,2 | <0.001 | 72,8 | <0.001 | 1,3 | ns | 3,6 | ns |
| Tree*N source | 1,4 | ns | 1,1 | ns | 1,4 | ns | 0,2 | ns |
| Tree*RH | 2,1 | ns | 2,1 | ns | 1,8 | ns | 1,9 | ns |
| N source*RH | 2,2 | ns | 0,3 | ns | 2,2 | ns | 0,03 | ns |
| Tree*N source*RH | 0,8 | ns | 1,1 | ns | 1,5 | ns | 0,9 | ns |

**Table 2.** The average and standard errors of fine root morphological traits (AD—average diameter; SRA—specific root area; SRL—specific root length; BI—branching intensity at moderate (mRH) and elevated relative air humidity (eRH) treatments of *Populus× wettsteinii*, *Betula pendula*, *Pinus sylvestris*, *Picea abies* and *Tilia cordata*. Lowercase letters indicate the trait significance comparing humidity treatment of each tree species (P < 0.05).

|  | *Populus× wettsteinii* | | *Betula pendula* | | *Pinus sylvestris* | | *Picea abies* | | *Tilia cordata* | |
| --- | --- | --- | --- | --- | --- | --- | --- | --- | --- | --- |
|  | mRH | eRH | mRH | eRH | mRH | eRH | mRH | eRH | mRH | eRH |
| AD (mm) | 0.3±0.04 a | 0.3±0.06 a | 0.2±0.02 a | 0.2±0.02 a | 0.5±0.03 b | 0.6±0.02 a | 0.6±0.04 a | 0.6±0.03 a | 0.3±0.05 a | 0.3±0.02 a |
| SRA (m² kgˉ¹) | 77±9 a | 92±11 a | 121±7 b | 170±14 a | 66±5 a | 52±3 b | 36±3 a | 44±3 a | 61±7 a | 68±7 a |
| SRL (m gˉ¹) | 102±28 a | 133±39 a | 198±22 a | 274±39 a | 50±7 a | 31±2 b | 21±3 a | 26±3 a | 70±14 a | 77±11 a |
| RTD (kg mˉ³) | 199±21 a | 184±31 a | 168±14 a | 118±9 b | 138±3 a | 144±6 a | 195±10 a | 168±7 b | 227±13 a | 213±15 a |
| BI (mgˉ¹) | 37±10 a | 56±22 a | 72±9 a | 82±14 a | 20±3 a | 12±1 b | 10±2 a | 12±2 a | 44±9 a | 46±8 a |
